# Exploring the Relationship of Comorbidities, Smoking Status, HRCT Findings With COVID-19 Disease Severity and Outcomes

**DOI:** 10.7759/cureus.52937

**Published:** 2024-01-25

**Authors:** Sohail Khan Raja, Rubina Rafique Shiekh, Mohammad Ali Arshad Abbasi, Samia Tariq, Humayun Saleem, Maham Tariq, Amna Akbar, Sarosh Khan Jadoon, Sabahat Tasneem, Mohammad Saleem Khan

**Affiliations:** 1 Pulmonology, Azad Jammu Kashmir Medical College, Muzaffarabad, PAK; 2 Medicine, Azad Jammu Kashmir Medical College, Muzaffarabad, PAK; 3 Medicine, Women Medical and Dental College Abbottabad, Abbottabad, PAK; 4 Public Health, Health Services Academy, Islamabad, PAK; 5 Radiology, Gujranwala Teaching Hospital, Gujranwala, PAK; 6 Emergency and Accident, District Headquarters Hospital (DHQ), Jhelum, PAK; 7 General Surgery, Combined Military Hospital, Muzaffarabad, PAK; 8 Medicine, District Headquarters Hospital (DHQ), Kotli, PAK

**Keywords:** disease severity, high resolution computed tomography, smoking, comorbid, covid-19 outcomes

## Abstract

Introduction: Coronavirus disease 2019 (COVID-19) is a serious illness that can affect multiple organs including the lungs. The COVID-mortality risk is attributed to the quick transmission of the virus, the severity of disease, and preclinical risk factors, such as the presence of comorbidities. High-resolution computed tomography (HRCT) can predict disease severity in COVID-19 patients.

Methodology: This was a retrospective cohort study in which data were obtained from COVID centers at tertiary care hospitals in Azad Jammu and Kashmir. Details of clinical characteristics and HRCT findings along with details of smoking and comorbid history were obtained.

Results: Fever at hospital admission, HRCT findings, and having a partner predicted disease severity showed a significant p-value of <0.05. Old age and living in a combined household were associated with severe outcomes (p<0.05). Symptoms of shortness of breath (SOB) on hospital admission could predict the need for ICU admission in COVID-19 patients.

Conclusion: HRCT has a good predictive value for disease severity in patients with COVID-19, and old age is a risk factor. Although, limited associations were established in the analysis, in this study hyperlipidemia and hypertension significantly affected the course of disease. Further studies should be done to explore the relationship.

## Introduction

The coronavirus disease 2019 (COVID-19) has emerged as a serious global health crisis and a substantial contributor to global patient mortality [1]. COVID-19 can affect the respiratory, digestive, urinary, hepatic, and central neurological systems in addition to the lungs. In addition to its immediate consequences, COVID-19 may also have long-term repercussions [2]. The high risk of mortality associated with COVID-19 is partly due to the virus’s propensity for rapid spread. The severity of illness in some people is another important factor that significantly affects mortality in COVID-19 patients [3]. Comorbidities are crucial for the development of severe diseases in COVID-19 patients [4,5]. Recent epidemiological data have suggested a counterintuitive relationship between smoking and contracting SARS-CoV-2 infection. Smoking appears to reduce the risk of contracting an infection [6]. Smoking history is linked to a lower likelihood of getting COVID-19, but it cannot be regarded as complicated by multiple comorbidities, and it is crucial for clinical guidance to categorize and focus on the effects of different comorbidities on pertinent clinical indicators and the prognosis of patients with COVID-19 [7].

This retrospective cohort study aimed to determine the association among smoking status, high-resolution computed tomography (HRCT) findings, disease severity, and outcomes. This study aimed to evaluate how these variables interact with and affect the course and prognosis of the illness. This study aimed to determine the predictive efficacy of CT scores in COVID-19 patients. This research aimed to offer a more systematic and objective assessment of lung involvement in COVID-19 cases; thus, they decided to quantitatively evaluate the CT data. The results of this study will improve our understanding of how smoking and concomitant conditions affect disease severity and offer useful information for improved management and clinical decision-making.

## Materials and methods

This study aimed to assess if there is an association between COVID-19 disease severity and the presence of a comorbid and if the outcomes are related to how the disease manifested on high-resolution computed tomography (HRCT) findings. This study was conducted at a tertiary care facility in Azad Jammu and Kashmir, Pakistan, with a sizable patient population and it was a center dedicated to COVID-19 patients during the pandemic. The data was collected retrospectively from May 2020 to May 2023 making the study span of three years during the pandemic. In this study, we only included patients who tested positive for COVID-19, which was determined through real-time reverse transcriptase polymerase chain reaction (rRT-PCR). Therefore, we refer to them as COVID-19 patients in the text. Patient confidentiality and privacy are rigorously upheld when gathering and analyzing COVID-19 data. Given the retrospective nature of the study, informed consent was not required. Permission for the study was granted by the ethical committee of the institute.

The following information was gathered for each participant: demographics like age, gender/sex, household type, type of residence (rural/urban), and medical background (previous history of disease). We wanted to measure the prevalence of the outcomes exclusively associated with COVID-19 and comorbid. So, common comorbid conditions, such as hypertension, diabetes, ischemic heart disease, chronic obstructive pulmonary disease, and smoking, were recorded. Clinical results, including hospitalization, the necessity for critical care, and the requirement for mechanical breathing, referral, and mortality, were saved. The frequency of symptoms was noted down. The common symptoms of COVID-19 were fever, cough, loss of taste, shortness of breath, GIT (gastrointestinal track) symptoms, and pain (head, muscle) or lethargy. Body mass index (BMI), pulse rate, systolic and diastolic pressure (SBP, DBP) were noted down and the course of disease at the hospital and prior to admission were all recorded.

The clinical characteristics and pulmonary involvement were recorded as markers of disease severity. It was categorized by the clinical parameters as mild, moderate, or severe. The presence of the following parameters defined the severity: a value of 300 mg or less of PaO_2_ (oxygen concentration or partial pressure of arterial blood oxygen), a respiratory rate (RR) of 30 beats/minute or more causing respiratory distress or shortness of breath, and resting blood oxygen saturation of 93% or less. The severity score from 1 to 5 was determined by the presence of these clinical presentations. Respiratory failure or critical condition was defined as the need for mechanical ventilation and/or other organ failure that needed hospital admission.

Patterns, presence of ground-glass opacities, consolidation, crazy paving patterns, fibrosis, etc. were typical and common HRCT findings in COVID-19. According to radiologyassistant.nl, coronavirus disease 2019 reporting and data system (CO-RADS) 1 to CO-RADS 6 are defined as normal, low probability of COVID-19, intermediate probability of COVID-19, high suspicion of COVID-19, typical COVID-19, and PCR+ve COVID-19. The readings of chest x-rays and HRCT were noted down and their values were evaluated by radiologist and codes to tabulate it in numeric form were designed by the radiologist like 0 was taken as no involvement, 1 as one side, and 2 as both lungs involvement. Follow-up data (length of follow-up, type of treatment, disease progression, or resolution) and follow-up HRCT findings were tabulated. The entries with complete data either on hospital sheets (hard copy) or on database (soft copy) were included in the study. The criteria for inclusion were the availability of hospital records, specifically HRCT availability. This means that only the data of those patients with a complete history of clinical findings and symptoms, as well as the availability of a high-resolution computed tomography scan, were included. The variables with missing values were not counted like cerebrovascular system (CVS), Parkinson’s disease, and Crohn’s disease were mentioned in very few patients. Patients with missing data were excluded. Terminal illnesses like cancer, liver cirrhosis, hepatic/renal failure, and conditions compromising the immune system like systemic lupus erythematosus and AIDS were excluded as these conditions were associated with a very high score of complications and could overwhelm the overall outcomes that we want to study.

The data was gathered on an Excel sheet by authors who volunteered for data curation and then it was transferred to SPSS 25 (Armonk, NY: IBM Corp.). Frequency and percentages of symptoms and all categorical variables including CO-RADS 1 to CO-RADS 6 were tabulated. Mean values with standard deviation were calculated for age, BMI, pulse, respiratory rate (RR), SBP, DBP, and SpO_2_. The chi-square test was used to determine the significance of categorical variables and the Independent Samples Kruskal-Wallis test was used to determine the significance of continuous variables. Regression analysis was also used to determine the correlation. A p-value of less than 0.05 was considered significant.

## Results

The prevalence of each comorbid condition and all risk factors were determined using descriptive analysis. The association between disease events and severity was determined through contingency analysis using the chi-square test. Multivariable regression models were used to assess the relationship between comorbidities, smoking status, HRCT findings, disease severity, and clinical outcomes while controlling for confounding factors (Table 1).

**Table 1:** Demographic variables and comorbidities.

Variable		Frequency	Percentage
Gender	Male	601	65.7%
	Female	314	34.3%
Household type	Joint	693	75.7%
	Nuclear	222	24.3%
Marital status	Single	432	47.2%
	Married	483	52.8%
Hypertension (HTN)	Non-HTN	363	39.7%
	HTN	552	60.3%
Diabetes mellitus (DM)	No-DM	555	60.7%
	DM	360	39.3%
Ischemic heart disease (IHD)	No IHD	767	83.8%
	IHD	148	16.2%
Chronic obstructive pulmonary disease (COPD)	No COPD	841	91.9%
	COPD	74	8.1%
Other comorbidities	No comorbidity	775	84.7%
	Comorbidity	140	15.3%
Smoking	No	747	81.6%
	Yes	168	18.4%

High temperature (72.9%), cough (68.3%), and dyspnea (66.2%) had the highest prevalence (Table 2). The need for ICU admission was 68.3% (n=628), and 84.5% (n=773) of the patients were discharged after symptom resolution (Table 3). A total of 55.1% of the patients had exhibited no findings in chest x-ray. Chest x-rays showed no typical signs of disease or lung involvement. High-resolution CT predicted mild-to-severe changes in all the PCR-positive patients (Table 4).

**Table 2:** Symptoms of COVID-19 disease in the present cohort. SOB: shortness of breath; GIT: gastrointestinal track; COVID-19: coronavirus disease 2019

Variable		Frequency	Percentage
Fever	Absent	248	27.1%
	Present	667	72.9%
Cough	Absent	290	31.7%
	Present	625	68.3%
SOB	Absent	309	33.8%
	Present	606	66.2%
Muscle ache	Absent	718	78.5%
	Present	197	21.5%
Headache	Absent	866	94.6%
	Present	49	5.4%
Sore throat	Absent	855	93.4%
	Present	60	6.6%
Runny nose	Absent	873	95.4%
	Present	42	4.6%
Chest pain	Absent	828	90.5%
	Present	87	9.5%
GIT upset	Absent	801	87.5%
	Present	114	12.5%
Lethargy	Absent	834	91.1%
	Present	81	8.9%

**Table 3:** Disease severity and outcomes.

Variable		Frequency	Percentage
ICU admission	No	287	31.4%
	Yes	628	68.6%
Disease severity	Mild	231	25.2%
	Mild-to-moderate	163	17.8%
	Moderate	287	31.4%
	Moderate-to-severe	155	16.9%
	Severe	65	7.1%
	Critical	14	1.5%
Outcome	Discharged	773	84.5%
	Deceased	110	12.0%
	Referred	32	3.5%

**Table 4:** Chest x-ray and HRCT findings. HRCT: high-resolution computed tomography; CO-RADS: coronavirus disease 2019 reporting and data system

Variable		Frequency	Percentage
Chest x-ray	No findings	504	55.1%
	Unilateral infiltration	219	23.9%
	Bilateral infiltration	192	21.0%
HCRT	CO-RADS 1	102	11.1%
	CO-RADS 2	68	7.4%
	CO-RADS 3	97	10.6%
	CO-RADS 4	412	45.0%
	CO-RADS 5	167	18.3%
	CO-RADS 6	69	7.5%

**Figure 1:**
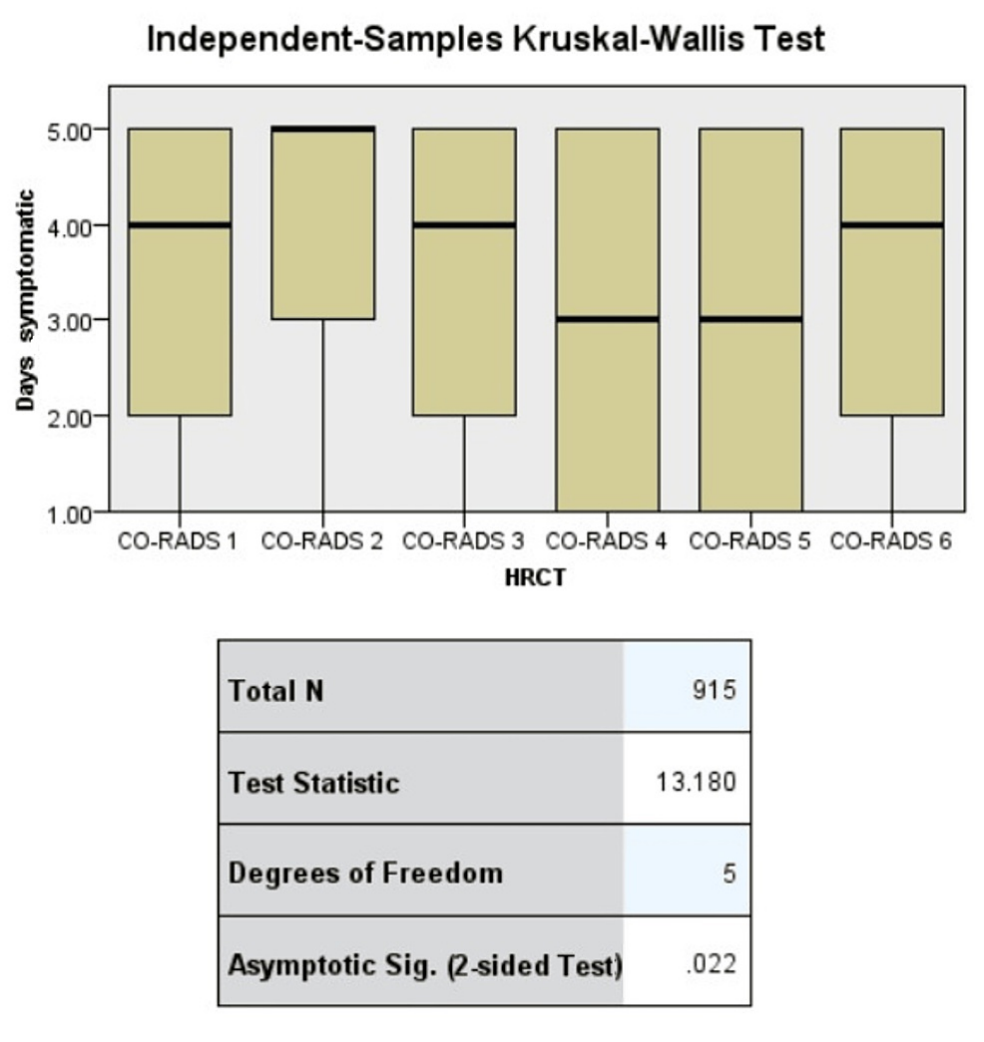
Symptoms of COVID-19 disease. COVID-19: coronavirus disease 2019; HRCT: high-resolution computed tomography; CO-RADS: coronavirus disease 2019 reporting and data system

**Figure 2:**
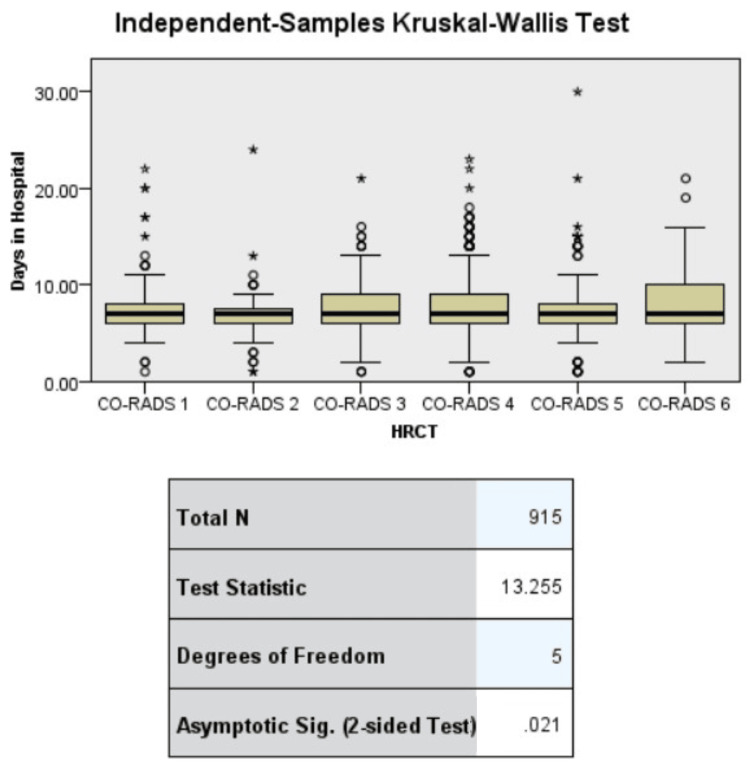
Association of high-resolution computed tomography with (a) symptomatic days and (b) days in hospital. HRCT: high-resolution computed tomography; CO-RADS: coronavirus disease 2019 reporting and data system

The mean value for age was 58.78±10.52 years and the mean BMI was 27.48 kg/m^2^ with a standard deviation of 3.82 (Table 5). Important associations found in the chi-square test were fever with disease severity, household outcomes, and shortness of breath on ICU admission. HRCT findings were associated with disease severity. Regression analysis confirmed the association of outcomes with household and disease severity, and ICU admission was associated with the initial symptoms (shortness of breath {SOB}) (Tables 6, 7). The HRCT findings were related to longer hospital stays and more days with active symptoms (Figures 1, 2).

**Table 5:** Means and standard deviation for continuous variables. *Mean to find dispersion and central tendency. RR: respiratory rate; SpO_2_: oxygen saturation

Variable	Median	Mean	SD
Age	59.00	58.78	10.52
BMI	27.2	27.48	3.82
Pulse	86	88.09	14.529
RR	26	26.81	5.797
Systolic pressure	123	126.99	16.984
Diastolic pressure	80	78.89	11.176
SpO_2_	93	90.28	8.069
Symptomatic days	4	3.27	1.680

**Table 6:** Association of variables with disease severity and outcomes. IHD: ischemic heart disease; COPD: chronic obstructive pulmonary disease; BMI: body mass index; HRCT: high-resolution computed tomography; SOB: shortness of breath

Variables	Disease severity	ICU admission	Outcomes
Gender	0.729	0.261	0.492
Age	0.411	0.460	0.001
BMI	0.377	0.065	0.377
Marital status	0.019	0.101	0.082
Household	0.708	0.372	0.029
Hypertension	0.076	0.546	0.575
Diabetes mellitus	0.284	0.670	0.158
IHD	0.517	0.280	0.836
COPD	0.339	0.837	0.109
Other comorbid	0.160	0.078	0.060
Smoking	0.363	0.955	0.513
Fever	0.025	0.293	0.482
Cough	0.673	0.281	0.462
SOB	0.116	0.006	0.949
Muscle ache	0.774	0.406	0.230
Headache	0.844	0.907	0.064
Sore throat	0.610	0.272	0.642
Runny nose	0.948	0.459	0.270
Chest pain	0.555	0.678	0.895
GIT upset	0.532	0.054	0.275
Lethargy	0.509	0.249	0.147
Chest x-ray	0.095	0.684	0.456
HRCT	0.018	0.345	0.082

**Table 7:** Regression analysis for outcomes and disease severity. *(a) ICU admission correlation with SOB. **Adjusted for gender. ***(b) Disease severity correlation with SOB. ****(c) Outcome correlation with disease severity and household. SOB: shortness of breath

Variables	Sig.	OR	95% CI for EXP(B)	Sig.	aOR	95% CI for EXP(B)
SOB*	0.007	1.496	1.119-2.000	0.007	1.488**	1.113-1.991
(a) ICU admission need						
SOB***	0.045	8.190	1.046 - 64.145	0.042	0.119**	0.015-0.930
(b) Severity of disease course						
Household	0.030	1.679	1.051-2.680	0.029	1.683	1.054-2.688
Disease severity	0.000	0.650	0.564-0.749	0.000	1.544	1.339-1.781
(c) Final outcome of the disease course****						

## Discussion

Among the COVID-19 patients, only 1.5% (n=14) developed critical symptoms, and 65 (7.1%) patients developed severe symptoms. Male sex was more prevalent, and the most prevalent symptoms were fever, shortness of breath, and cough. Previous studies have shown that the presence of comorbidities alters the course of COVID-19. Hyperlipidemia and hypertension were the most common comorbid conditions, while chronic diabetes, being overweight, and having mental illness were the most significant risk factors [8]. Several factors contribute to the association between hypertension and COVID-19 severity. First, people with hypertension frequently have underlying medical illnesses, including diabetes and cardiovascular disease, which are known to be linked to an increased risk of developing severe COVID-19. The SARS-CoV-2 virus may enter and replicate more easily as a result of this vascular dysfunction, increasing the severity of the infection [9]. Older patients are more vulnerable to and at risk of COVID-19. Their increased vulnerability is a result of age-related immune system changes, underlying medical problems, respiratory system abnormalities, and social factors [10]. Long-term medication for the treatment of comorbidities affects the cascade of COVID-19 symptoms and reduces the number of therapeutic options for the treatment of COVID-19 [11]. The ability of CT scans to identify recognizable lung abnormalities, such as solidified lung tissue, ground glass opacities, and intersecting line patterns, has made them increasingly accepted for COVID-19 diagnosis and prognosis. However, not much research has been conducted on the importance of CT results in predicting disease severity [12]. The severity of symptoms is probably not the criterion for predicting the outcomes of the course of COVID-19, as is the number of patients who develop severe or critical symptoms. We had to make 32 referrals to local hospitals for patients who required further hospital care and treatment (Table 3). The lung involvement was defined as COVID-19 Reporting and Data System (CO-RADS). There are six categories of CO-RADS with CO-RADS 1 in which the lowest probability of COVID has already been established. CO-RADS 4 accounted for 45% of all findings, and CO-RADS 5 accounted for 18.3%. The HRCT findings predicted COVID-19 in all patients with high susceptibility. The CO-RADS 6 score was present in 69 patients, which indicated a confirmed diagnosis. Higher HRCT scores predicted more severe diseases (p=0.018). The positive association between HRCT-determined CO-RDS and disease severity indicates the importance of an HRCT-based diagnosis in COVID-19 patients (Table 4). HRCT values were associated with disease severity in the present study (p=0.018), as reported in a previous study in which patients with severe or critical conditions acquired higher CT scores as the disease progressed. The patients’ second-week CT scores demonstrated a predictive value for disease severity [12]. HRCT value was tested against the number of days the patient remained symptomatic and the number of days the patient stayed at the hospital. In the present study, HRCT could predict the number of days a patient would suffer active symptoms and also stay at the hospital (p<0.05). We can say that high-resolution computed tomography can be used for determining disease duration (measured as number of days symptomatic and days stayed at hospital) (Figure 2). Hypertension is a unique predictor of severe COVID-19 and has significant effects on clinical care and public health initiatives [9]. However, this association was not observed in the present study. Monitoring hypertensive COVID-19 patients carefully should be included in the protocol. The presence of fever is associated with the development of disease severity, which means that in patients with high fever, there is a chance that they will be critical. Shortness of breath was linked to the need for admission to the ICU, and a joint family system or shared household was associated with the outcomes. People living in joint families had worse outcomes than those living in isolated homes or nuclear family systems (Table 6). Elderly people are more susceptible to COVID-19, which has important ramifications for the healthcare and public health systems. Regarding the diagnosis and treatment of COVID-19, healthcare professionals must be aware of the special requirements of senior patients. Early detection and prompt care are essential to reduce the risk of serious illnesses in the population. Elderly COVID-19 patients can benefit from close symptom monitoring, routine health checks, and quick medical interventions to reduce risk and improve results [10]. Similar to the results of the present study, elderly patients had poor outcomes. Prolonged hospital stays and acute critical conditions were predicted in the critical group. Coexisting diseases and psychiatric disorders were also found to be predictive. Adults aged <60 years with coexisting disease and sudden severe sickness had a higher likelihood of manifesting physical symptoms in the moderate group, whereas adult women with longer hospital admissions were more likely to experience intellectual and psychiatric disabilities [13]. In the present study, outcomes were associated with age but not with any comorbidities. Disease severity predicted outcomes, even when adjusted for sex (Table 7). A binary logistics regression was conducted to confirm the association of variables that showed significant p-value in the chi-square test. SOB was associated with the need for ICU admission, and the associations remained significant even after adjusting for sex (OR=1.496 and aOR=1.488). Although the majority of COVID-19 cases only show mild-to-moderate symptoms, a sizable minority of patients have severe illness. Pneumonia, multiorgan failure, and acute respiratory distress syndrome (ARDS), which can be fatal, are common symptoms in severe cases. Elderly people and those with underlying medical disorders, such as cardiovascular disease, diabetes, and respiratory illnesses, are especially vulnerable to severe forms of the illness, resulting in increased fatality rates within this population [3]. In the present study, comorbid conditions had no effect on the prognosis of COVID-19 patients. Patients with prior respiratory disease had significantly higher mortality, greater need for ICU admission, and greater need for ventilator support.

Potential confounders, including age, sex, race, BMI, and 10 common comorbidities, were considered [4]. COPD had no effect on the outcomes, but shortness of breath was associated with the need for ICU admission and final outcomes. Smoking history is predictive of a lower likelihood of contracting COVID-19; however, it cannot be considered a protective factor because smokers develop more severe symptoms once they have contracted the disease [6]. However, no such results were obtained in this study. The results of a previous study demonstrated that former smokers were hospitalized and died from COVID-19 at a much higher rate than current smokers or non-smokers. Age and comorbidities among former smokers mediate this effect [14], as confirmed in another study [15]. Current tobacco use was linked to a decreased hazard of both SARS-CoV-2 infection and critical COVID-19 disease in a cohort analysis of 2.4 million persons after controlling for sociodemographic and medical comorbidities. Comparing never-smokers with those who had smoked in the past, it was discovered former smokers had higher death rates, advanced respiratory support needs, and mechanical ventilation needs than non-smokers; these differences were strongly mediated by smoking-related comorbidities. It is important to examine how the observed COVID-19 risk is lower in current smokers than in never smokers. Vaccine outreach and therapies as they become available should prioritize people with smoking-related comorbidities, according to the results [16,17]. A multiethnic Asian cohort of patients of ST-elevation myocardial infarction (STEMI) and non-ST elevation myocardial infarction (NSTEMI) was used to establish the existence of a smoker’s pseudo-paradox and to describe an elevated incidence of recurrent MI, although not mortality, among smokers [18]. However, no smoking associations were established in the study population.

Age, sex, and the type of comorbidities may all be factors posing a high risk for critically ill COVID-19 patients, according to the most recent epidemiological data [7]. Other important hazards for COVID-19 patients reported in previous research include diabetes, high blood pressure, respiratory illness, and cerebrovascular and cardiac problems.

Effective and early clinical evaluation and management of individuals with COVID-19 can be aided by doctors’ knowledge of these risk factors [19]. The presence of comorbidities may increase the chances of poor prognosis in COVID-19 patients. Patients who have a higher load of vascular risk factors have a greater risk of developing severe COVID-19, and they may benefit from early hospital treatment and specialized COVID-19 preventative strategies (such as self-isolation) [20]. Age directly increased the severity of COVID-19. The critical disease group was characterized by multiorgan injury, dyspnea, and a sustained inflammatory response. The overall mortality rate in the critical group was more than 85%, which was higher than that in the severe disease group (up to 58%) and moderate disease group (up to 18%), while the present study only indicated age as a risk factor [1].

Risk factors associated with fatal and critical outcomes related to COVID-19 include chronic comorbidities, such as diabetes, excessive weight, high blood pressure, cardiac and renal dysfunction, respiratory issues, and cancer. Excessive weight is the most prevalent respiratory illness with the strongest predictive value [21]. However, no such association was observed in this study.

Shortness of breath and disease severity coexisted (OR=8.19), and when adjusted for sex (aOR=0.119), the odds ratios were different. This indicates that sex enhances the effect of SOB symptoms on disease severity. Disease outcomes were also independent of sex when the association was tabulated against household type. A joint family system or living in a shared household had greater odds of developing the disease (OR=1.67 and aOR=1.68), and this was not dependent on the sex of the patient. Disease severity and outcomes were strongly associated (OR=0.564) and exaggerated when adjusted for sex (aOR=1.54), but the association remained positive and high in both cases (0.000). The main risk factors identified in this study were age, joint family system, or shared household. The most alarming symptom on admission was shortness of breath. Patients with these symptoms on admission are prone to poor outcomes and develop severe conditions.

Limitations

This is a retrospective cohort study; the researchers could not include all the COVID-19 patients in it because of missing data. The associations were very limited. All comorbid conditions were not associated in the same manner.

## Conclusions

Through the present study, researchers tried to explore the clinical course of COVID-19 patients who tested positive via PCR test. Disease manifestation and progression are exhibited as severity and duration of symptoms. Among this cohort of COVID-19 patients, a small proportion of patients developed critical illness and the overall prognosis was not bad. Comorbidities that played a role in shaping the disease’s trajectory were hyperlipidemia and hypertension. The elderly in this cohort were more vulnerable as reported by previous studies. Smoking did not follow the anticipated relationship with disease severity. Lastly, the study emphasizes the role of HRCT in diagnosing COVID-19 pneumonia.
